# The Usefulness of Falls Efficacy Scale-International in Predicting Falls in Chronic Kidney Disease Patients

**DOI:** 10.3390/jcm15166455

**Published:** 2026-08-20

**Authors:** Patryk Jerzak, Mariusz Kusztal, Krzysztof Benc, Wioletta Dziubek, Łukasz Rogowski, Bożena Ostrowska, Maja Pieczaba, Maciej Gołębiowski, Wiktoria Kłosowska, Anna Wiśniewska, Mirosław Banasik, Tomasz Gołębiowski

**Affiliations:** 1 Department of Nephrology, Transplantation Medicine and Internal Diseases, Institute of Internal Diseases, Wroclaw Medical University, 50-556 Wroclaw, Poland; mariusz.kusztal@umw.edu.pl (M.K.); krzysztof.benc@umw.edu.pl (K.B.); maja.pieczaba@umw.edu.pl (M.P.); miroslaw.banasik@umw.edu.pl (M.B.); tomasz.golebiowski@umw.edu.pl (T.G.); 2 Faculty of Physiotherapy, Wroclaw University of Health and Sport Sciences, 51-612 Wroclaw, Poland; wioletta.dziubek@awf.wroc.pl (W.D.); bozena.ostrowska@awf.wroc.pl (B.O.); 3 Faculty of Health and Physical Culture Sciences, Witelon Collegium State University, 59-220 Legnica, Poland; rogowski.nwsm@wp.pl; 4 Faculty of Medicine, Wroclaw Medical University, 50-367 Wroclaw, Poland; maciej.golebiowski@student.umw.edu.pl (M.G.); wiktoria.klosowska@student.umw.edu.pl (W.K.); kontakt.annawis@gmail.com (A.W.)

**Keywords:** motor skills disorders, accidental falls, chronic kidney disease

## Abstract

**Background:** The Falls Efficacy Scale-International (FES-I) is an assessment tool designed to measure the level of fear of falling in older adults. This scale was developed to evaluate how much an individual fears falling during various daily activities. The aim of the study was to assess the discriminatory power of FES-I to predict falls within 2 years. The secondary objective was to evaluate the relationship between FES-I and vascular status. **Material and Methods:** In this prospective study, 130 patients (mean age, 64.7 ± 14.6 years) with chronic kidney disease (CKD) were analyzed. Of these, 90 patients had nondialysis CKD: 15 had stage G3 (G3a, n = 5; G3b, n = 10), 38 had stage G4, and 37 had stage G5. The remaining 40 patients (30.7%) were prevalent patients receiving maintenance hemodialysis. The most frequent cause of CKD was hypertension and diabetes in 57 (43.8%) of patients. The Falls Efficacy Scale-International (FES-I) was used to assess fear of falling. The Charlson Comorbidity Index (CCI) was used to measure comorbidity, and the 10-year cardiovascular risk was assessed using a web-based calculator QRESEARCH Cardiovascular Risk Algorithm, version 3 (QRISK^®^3), and hemodynamic parameters were measured using a Mobil-O-Graph monitor. Participants were followed for 2 years to assess the occurrence of falls. **Results:** During the two-year follow-up, 49 of 130 participants (37.7%) experienced at least one fall. FES-I demonstrated good discrimination for falls (AUC 0.836; bootstrap 95% CI, 0.760–0.903). In multivariable logistic regression adjusted for age, sex, dialysis status, comorbidity, and functional status, FES-I remained independently associated with falls (adjusted OR per 1-point increase, 1.145; 95% CI, 1.060–1.237; *p* < 0.001). Addition of FES-I to the basic clinical model increased the AUC from 0.826 to 0.874, although the difference was of borderline statistical significance in paired receiver operating characteristics (ROC) comparison (*p* = 0.053). The data-derived Youden-optimal threshold was 25 points; however, bootstrap analysis indicated threshold variability, and this cut-off should be considered exploratory. **Conclusions:** Higher FES-I scores were independently associated with falls during two-year follow-up and may provide additional prognostic information beyond selected clinical risk factors. FES-I may represent a candidate screening instrument for fall-risk assessment in CKD; however, the proposed cut-off (25 points) requires prospective external validation.

## 1. Introduction

Falls represent a major clinical concern among patients with chronic kidney disease (CKD), contributing significantly to morbidity, mortality, and reduced quality of life. According to epidemiological data, compared to the general population, CKD is independently linked to an increased risk of falls [1]. Furthermore, observational studies have shown that falls occur more frequently in CKD patients and are often associated with serious complications, including fractures and hospitalizations [2]. Even after controlling for comorbid conditions and lifestyle factors, older people with CKD still have a higher risk of fall-related injuries [3]. Significantly, recurrent falls have also been found to be an independent predictor of mortality in older dialysis patients [4].

Falls in CKD are multifactorial and are associated with polypharmacy, orthostatic hypotension, anemia, sarcopenia, metabolic disturbances, cognitive decline, frailty, and reduced mobility [5,6,7,8]. Neuromuscular dysfunction, including impaired peripheral sensation, muscle weakness, and reduced physical performance, may further compromise balance and coordination [9]. In patients receiving hemodialysis, reduced muscle strength and impaired physical performance have been identified as important predictors of falls, and fall incidence is higher than that reported in community-dwelling older adults [10,11]. Objective tools such as the Physiological Profile Assessment (PPA), which integrates sensory, motor, and balance-related domains, can identify individuals at increased risk of falling [12].

Frailty is highly prevalent in CKD and is associated with adverse outcomes, including functional deterioration, hospitalization, and mortality [13,14]. However, comprehensive physiological assessment may require dedicated equipment and trained personnel, highlighting the need for simpler approaches to fall-risk screening in routine nephrology practice. The Falls Efficacy Scale-International (FES-I) provides a brief patient-reported assessment of concern about falling during daily activities. Nevertheless, its prospective predictive value in patients with CKD and its incremental value beyond readily available clinical risk factors remain insufficiently established.

Arterial stiffness is also common in CKD and contributes to the increased cardiovascular burden in this population [15,16]. It has been associated with cerebral microvascular injury and cognitive impairment [17,18,19,20,21], while elevated pulse wave velocity has been associated with fall risk in older adults [22]. These observations provide a rationale for exploring vascular status in relation to FES-I; however, vascular assessment represents a secondary and exploratory aspect of the present study.

Therefore, the primary aim of this study was to evaluate the discriminatory ability of FES-I for falls during a 2-year follow-up in patients with CKD and to assess whether FES-I provides prognostic information beyond selected clinical risk factors. The secondary objective was to explore the relationship between FES-I scores and vascular status, with particular emphasis on arterial stiffness parameters.

## 2. Materials and Methods

The final analysis of this prospective study included 130 patients with CKD (mean age, 64.6 ± 14.6 years; 54 females). 90 of these patients had nondialysis chronic kidney disease (CKD): 15 had stage G3 (G3a, n = 5; G3b, n = 10), 38 had stage G4, and 37 had stage G5. Prevalent patients undergoing maintenance hemodialysis made up the remaining 40 patients (30.7%). Dialysis vintage was not available because it was not methodically gathered. Potential participants were identified through a review of inpatients admitted between March 2021 and September 2021 for the diagnosis of CKD and vascular access creation.

Patients with CKD stage G3–5 had to meet three requirements in order to be included: (1) be at least eighteen years old; (2) be able to move around without the need of a wheelchair or cane; and (3) be able to give informed consent. Although FES-I was originally developed primarily for older adults, we included all adults with advanced CKD because functional impairment, frailty, and concern about falling may occur at younger chronological ages in this population. Age was therefore not used as an additional restriction but was accounted for in the multivariable analysis. The exclusion criteria were: (1) participation in another trial, (2) receiving emergency in-patient care within the past four weeks, and (3) inability to walk without additional aids. All patients participating in the study provided written informed consent.

**Figure 1:**
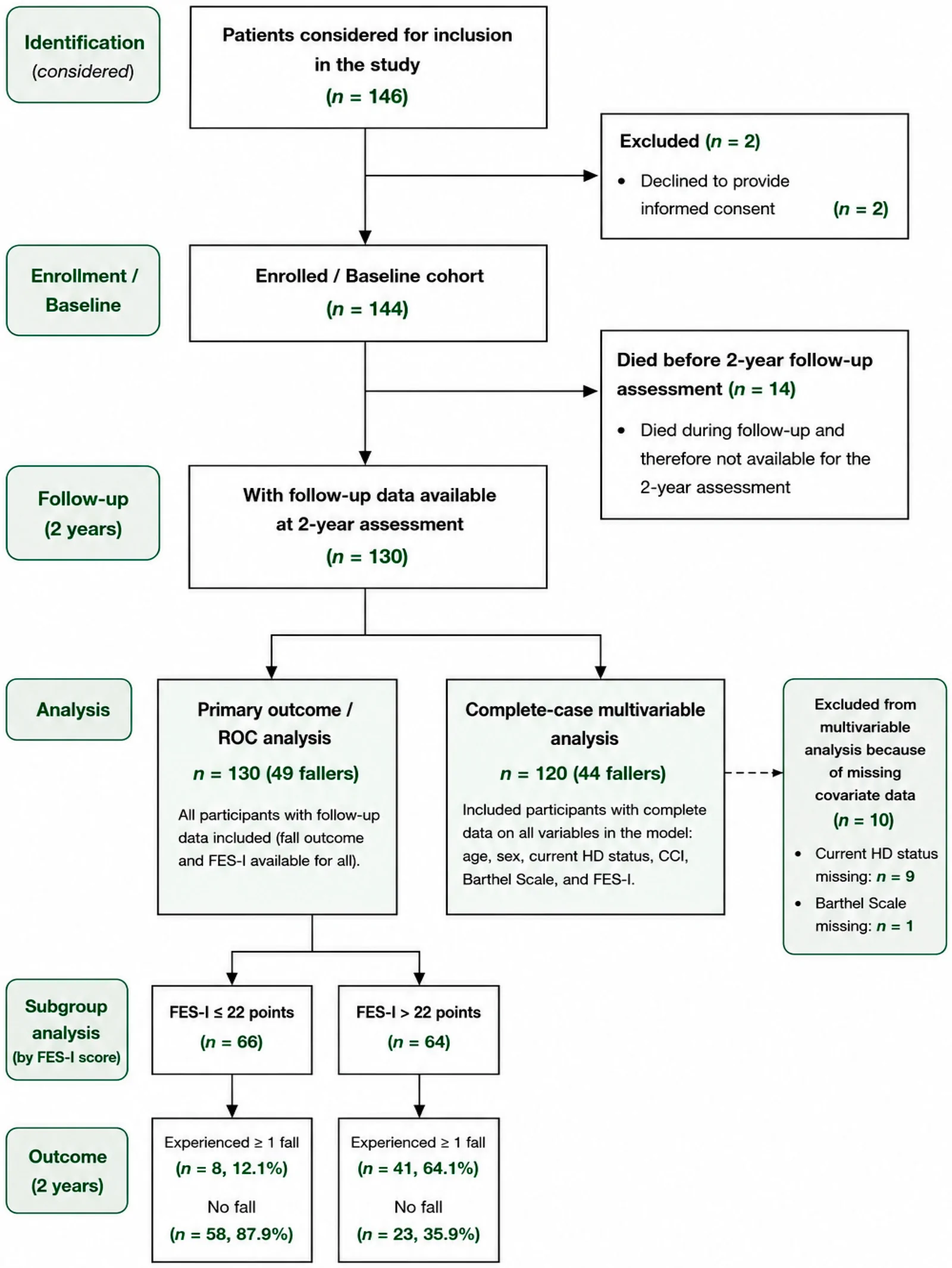
Participant flow diagram. FES-I, Falls Efficacy Scale-International; CCI, Charlson Comorbidity Index.

A total of 146 patients were initially considered for inclusion in the study. Two patients declined to provide informed consent and were therefore excluded. Of the 144 participants enrolled in the study, 14 died before the 2-year follow-up assessment and were therefore not included in the final analysis. Consequently, follow-up data were available for 130 participants, who constituted the final analytical cohort. Complete interview, questionnaire, and measurement data were available for the vast majority of these participants (Figure 1).

Concern about falling was assessed using the 16-item Falls Efficacy Scale-International (FES-I) [23,24]. Items are scored from 1 to 4, yielding a total score of 16–64, with higher scores indicating greater concern about falling. The validated Polish version was administered face-to-face by a trained investigator [25]. All items were required to calculate the total score; questionnaires with missing responses were excluded from the analysis.

The Charlson Comorbidity Index (CCI) was used to measure comorbidity load; higher scores denoted a higher overall burden of comorbid disease.

Fall-related physiological performance was assessed using the Short-Form Physiological Profile Assessment (S-PPA), comprising measures of visual contrast sensitivity, proprioception, quadriceps strength, reaction time, and postural sway. The component results were combined using the S-PPA software (Neuroscience Research Australia (NeuRA) FallScreen® assessments) to obtain a standardized composite fall-risk score (z-score), with higher values indicating greater physiological fall risk.

### 2.1. Visual Contrast Sensitivity

Visual contrast sensitivity was assessed using the Melbourne Edge Test and expressed in decibels (dB), with lower values indicating poorer contrast sensitivity.

### 2.2. Proprioception

Lower-limb proprioception was assessed using a matching task performed with the eyes closed. The alignment error between the lower limbs was measured in degrees and averaged across five trials.

### 2.3. Muscle Force Test

Quadriceps strength was measured using a digital dynamometer with resistance applied 10 cm above the ankle; the best of three trials was recorded in kilograms.

### 2.4. Reaction Time Test

Reaction time to a visual stimulus was measured in milliseconds using a hand-operated response switch; the best result from ten trials was recorded.

### 2.5. Balance Test

Postural sway was assessed with a sway meter positioned at the lower back while participants stood for 30 s with eyes open on a 15 cm foam mat. Anteroposterior and mediolateral displacement was recorded.

### 2.6. Supplementary Functional Assessment

In addition to the five standard components of the S-PPA, supplementary functional assessments were performed. These tests were not used as substitutes for, or components of, the S-PPA composite score. Lower-limb functional strength was assessed using the 30 s sit-to-stand test, with the total number of correctly completed repetitions recorded.

The QRISK^®^3 score was used to estimate the 10-year risk of myocardial infarction or stroke based on established demographic and clinical cardiovascular risk factors.

Hemodynamic parameters were assessed using the Mobil-O-Graph monitor (IEM, Stolberg, Germany). Measurements included brachial systolic and diastolic blood pressure, pulse pressure, cardiac output, ejection fraction, augmentation index normalized to a heart rate of 75 beats/min (AIx@75), and pulse wave velocity (PWV). PWV was used as an index of arterial stiffness. In patients receiving maintenance hemodialysis, all study assessments, including the FES-I, S-PPA, supplementary functional tests, and vascular measurements, were performed approximately 2 h before the subsequent hemodialysis session.

Falls ascertainment. The primary study outcome was the occurrence of at least one fall during the two-year follow-up period. The total number of falls was reported as a secondary descriptive outcome and was not included in inferential analyses. A fall was defined as “an unexpected event in which the participant comes to rest on the ground, floor, or lower level”, according to the World Health Organization definition. Information regarding falls was collected during the scheduled follow-up visit two years after the baseline assessment using a structured interview conducted by the study investigators. Participants were asked whether they had experienced one or more falls during the follow-up period and, if applicable, the approximate number of falls. No prospective fall diaries or scheduled interim contacts were used.

Standard software (Statistica Version 13.3, StatSoft, Tulsa, OK, USA) was used to conduct the statistical analysis. Depending on the normality of the variables determined by the Kolmogorov–Smirnov test, continuous variables between groups were reported as mean and standard deviation (±SD) and compared using the independent t-test or Mann–Whitney U test. The χ^2^ test was used to compare categorical variables that were reported as absolute (n) and percentage (%). The comparisons presented in Table 1 and the correlation analyses were considered exploratory. Given the multiple comparisons performed, no adjustment for multiplicity was applied, and the corresponding *p*-values were interpreted descriptively. The association between FES-I and PWV was assessed using Pearson’s correlation coefficient. As a sensitivity analysis less sensitive to outliers and departures from distributional assumptions, Spearman’s rank correlation was additionally calculated. Internal consistency of the FES-I was assessed using Cronbach’s alpha and McDonald’s omega, with 95% confidence intervals estimated using 2000 bootstrap resamples. Missing data were assessed separately for each variable included in the multivariable model. Data were complete for age, sex, Charlson Comorbidity Index (CCI), FES-I score, and fall outcome. Current hemodialysis status was unavailable for 9 of 130 participants (6.9%), and the Barthel Scale was unavailable for 1 participant (0.8%). Consequently, the primary multivariable logistic regression was performed as a complete-case analysis and included 120 participants, of whom 44 experienced at least one fall during the 2-year follow-up. To assess the robustness of the complete-case findings to missing covariate data, a sensitivity analysis using multiple imputation by chained equations was performed. Fifty imputed datasets were generated using the variables included in the multivariable model together with the fall outcome. The prespecified logistic regression model was fitted in each imputed dataset, and estimates were pooled using Rubin’s rules. Multivariable logistic regression was performed to evaluate whether FES-I was independently associated with the occurrence of at least one fall during the two-year follow-up. To limit model overfitting given the number of observed events, a parsimonious model with clinically prespecified covariates was used, including age, sex, current hemodialysis status, Charlson Comorbidity Index (CCI), Barthel scale, and FES-I score. Adjusted odds ratios (ORs) with 95% confidence intervals (CIs) were reported. Multicollinearity was assessed using variance inflation factors (VIFs). The incremental predictive value of FES-I was evaluated by comparing two nested logistic regression models fitted in the same complete-case analytical sample of 120 participants, including 44 participants who experienced at least one fall. The basic clinical model included age, sex, current hemodialysis status, Charlson Comorbidity Index (CCI), and Barthel Scale. The extended model additionally included FES-I. Apparent discrimination was quantified using the area under the receiver operating characteristic curve (AUC), and the AUCs of the nested models were compared using the paired DeLong test. Improvement in overall model fit after addition of FES-I was assessed using a likelihood-ratio test. Internal validation was performed using 2000 bootstrap resamples. In each bootstrap iteration, 120 participants were sampled with replacement from the complete-case analytical dataset, and each model was refitted de novo using the same prespecified predictors as in the original model. Model performance was evaluated in the bootstrap sample and, without further refitting, in the original analytical dataset. Optimism was calculated as the mean difference between bootstrap-sample and original-sample performance across all resamples and was used to obtain optimism-corrected estimates. Internal validation was applied to the AUC, Brier score, calibration intercept, and calibration slope. For the Brier score, for which lower values indicate better performance, the mean bootstrap-derived increase in prediction error was added to the apparent Brier score. The calibration intercept and slope were estimated by logistic recalibration of the observed outcome on the logit of the predicted probability. Apparent calibration intercept and slope were 0 and 1, respectively, by construction. For the univariable ROC analysis of FES-I, the threshold maximizing the Youden index was identified. Sensitivity, specificity, positive predictive value (PPV), and negative predictive value (NPV) were calculated with 95% confidence intervals. Bootstrap resampling was additionally used to assess the internal stability of the ROC performance and selected threshold. The primary inferential analysis evaluated the association between FES-I and the occurrence of at least one fall using multivariable logistic regression adjusted for clinically relevant covariates. ROC analysis was used to assess the discriminative performance of FES-I and to explore potential cut-off values. A *p*-value < 0.05 was considered significant.

## 3. Results

### 3.1. Baseline Clinical Data

The median FES-I score within the study group was twenty-two points. The FES-I demonstrated excellent internal consistency in the present CKD cohort (Cronbach’s α = 0.964, 95% CI 0.949–0.974; McDonald’s ω = 0.967, 95% CI 0.953–0.976). Based on this, we divided our cohort into two subgroups with FES-I scores of ≤22 and >22 points. The demographic baselines, cause of CKD, comorbidities, S-PPA test results, hemodynamic parameters, blood chemical features, questionnaires, and data from a two-year follow-up are all shown in Table 1. Kidney failure was mostly caused by diabetes and renovascular disease, with glomerulonephritis and other illnesses following. In the group of patients with a higher FES-I score > 22 points, diabetes and hypertension nephropathy occurred more frequently than in the group of patients with low FES-I, and conversely, glomerulonephritis as a cause of chronic kidney disease occurred more frequently in patients with FES-I ≤ 22 points. According to the QRISK3 survey, the group with a high score (FES-I > 22 points) was significantly older, had a higher BMI, had a higher CCI, and was more likely to have a heart attack or stroke within the following ten years. Comparing the results obtained in the Short Form Physiological Profile Assessment, we found a higher fall risk score (z-score and fall risk score (point)), lower ability to contrast discrimination in the Melbourne Edge Test, poorer proprioception, lower muscle strength in the 30 Second Sit to Stand Test and knee extension test, and greater postural swaying in the subgroup of patients with higher FES-I scores (>22 points). This subgroup is also characterized by a lower quality of life score on the SF-36 scale and greater problems with self-care and disability (Barthel score and CFS Rockwood score).

During the two-year follow-up, 49 of 130 participants (37.7%) experienced at least one fall. A total of 84 falls were recorded during follow-up. Among participants with FES-I ≤ 22, 8 patients experienced at least one fall, whereas 41 patients with FES-I > 22 experienced at least one fall. The total number of falls was 11 and 73 respectively.

**Figure 2:**
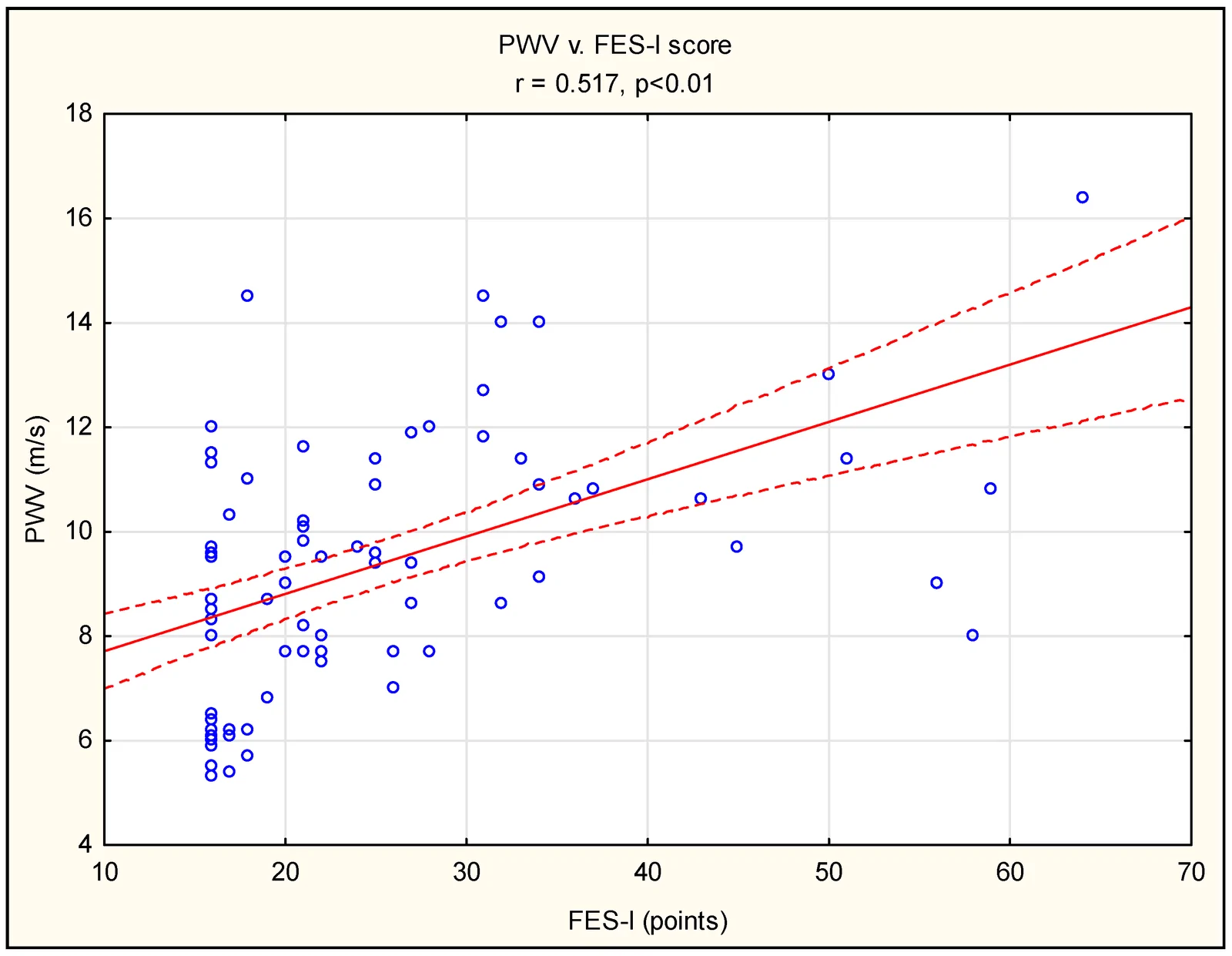
Pearson’s correlation analysis between PWV and FES-I. Abbreviations: PWV, pulse wave velocity; FES-I, Falls Efficacy Scale-International.

Patients with FES-I > 22 points had higher pulse wave velocity (PWV), a measure of arterial stiffness, and higher peripheral pulse pressure (pPP). In the exploratory correlation analysis, FES-I was moderately positively correlated with PWV (Pearson’s r = 0.441, *p* < 0.001). A sensitivity analysis using Spearman’s rank correlation yielded a similar association (ρ = 0.471, *p* < 0.001), supporting the robustness of this finding (Figure 2). Exploratory analyses also showed positive correlations between FES-I and both the S-PPA z-score (r = 0.58, *p* < 0.05) and fall-risk score (r = 0.39, *p* < 0.05).

### 3.2. Multivariable Predictors of Falls

To assess the independent association between FES-I and the incidence of at least one fall during the two-year follow-up, a parsimonious multivariable logistic regression model was built. Complete data for all variables included in the prespecified multivariable model were available for 120 of 130 participants (92.3%), including 44 of the 49 participants who experienced at least one fall. Ten participants were excluded from the complete-case analysis because of missing covariate data (current hemodialysis status, n = 9; Barthel Scale, n = 1). After adjustment for age, sex, dialysis status, CCI, and Barthel scale, FES-I remained independently associated with falls (adjusted OR per 1-point increase, 1.145; 95% CI, 1.060–1.237; *p* < 0.001). Age (OR 1.035; 95% CI, 0.980–1.092; *p* = 0.216), female sex (OR 2.437; 95% CI, 0.876–6.779; *p* = 0.088), dialysis status (OR 2.177; 95% CI, 0.771–6.147; *p* = 0.142), CCI (OR 1.149; 95% CI, 0.898–1.472; *p* = 0.269), and Barthel scale (OR 1.050; 95% CI, 0.976–1.130; *p* = 0.190) were not independently associated with the outcome in this model (Table 2). No substantial multicollinearity was detected; all VIF values were below 5, with the highest VIF observed for FES-I (3.31).

In a sensitivity analysis using multiple imputation for missing covariate data, the association between FES-I and falls remained essentially unchanged. The pooled adjusted OR for FES-I was 1.140 per 1-point increase (95% CI, 1.059–1.227; *p* < 0.001), compared with 1.145 (95% CI, 1.060–1.237; *p* < 0.001) in the complete-case analysis. Thus, the principal finding was robust to the method used to handle missing covariate data.

### 3.3. Predictive Performance and Incremental Value of FES-I

Both nested models were evaluated in the same complete-case analytical sample of 120 participants, including 44 fallers. The basic clinical model included age, sex, current hemodialysis status, CCI, and Barthel Scale, whereas the extended model additionally included FES-I. The apparent AUC was 0.826 for the basic model and 0.874 for the extended model, corresponding to an absolute increase of 0.048 after the addition of FES-I. The difference in AUC was of borderline statistical significance in the paired DeLong comparison (*p* = 0.0529). In contrast, the addition of FES-I significantly improved overall model fit according to the likelihood-ratio test (χ^2^ = 15.45, df = 1, *p* = 0.000085).

Following internal validation using 2000 bootstrap resamples, the optimism-corrected AUC was 0.798 for the basic model and 0.850 for the extended model. The apparent Brier scores were 0.161 and 0.131, respectively, and the corresponding optimism-corrected Brier scores were 0.181 and 0.151. The optimism-corrected calibration slopes were 0.833 for the basic model and 0.846 for the extended model, with corresponding calibration intercepts of −0.073 and −0.063. These findings indicate that the addition of FES-I improved overall model fit and was associated with better discrimination and prediction accuracy. However, the improvement in AUC did not reach the conventional threshold for statistical significance in the paired DeLong test, and the internally validated results should be regarded as exploratory pending external validation.

### 3.4. ROC Analysis of FES-I

**Figure 3:**
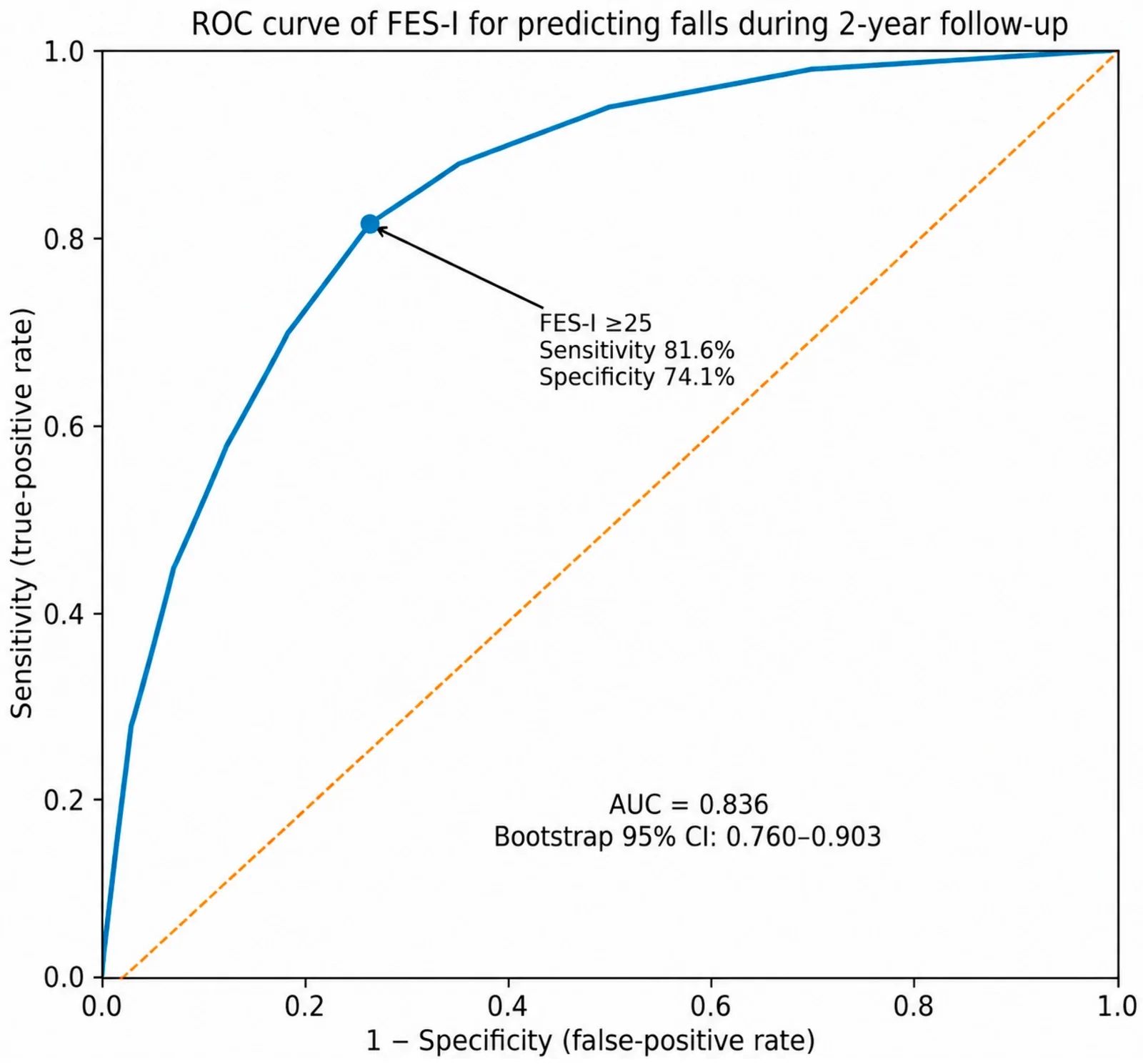
Receiver operating characteristic curve of the FES-I score for predicting at least one fall during the 2-year follow-up. The data-derived threshold of ≥25 points corresponded to a sensitivity of 81.6% and specificity of 74.1%. The threshold should be considered exploratory and cohort-specific.

In the complete cohort of 130 participants (49 fallers and 81 non-fallers), FES-I demonstrated good discrimination for the occurrence of at least one fall during the two-year follow-up, with an AUC of 0.836 (bootstrap 95% CI, 0.760–0.903). The threshold maximizing the Youden index in the present dataset was 25 points (Figure 3). Using FES-I ≥ 25 as a positive test, 40 of 49 fallers were correctly classified (true positives), whereas 9 were classified as false negatives; among 81 non-fallers, 60 were correctly classified as true negatives and 21 as false positives (Table 3). This corresponded to a sensitivity of 81.6% (95% CI, 68.0–91.2%), specificity of 74.1% (95% CI, 63.1–83.2%), PPV of 65.6% (95% CI, 52.3–77.3%), and NPV of 87.0% (95% CI, 76.7–93.9%) at the observed fall prevalence of 37.7%. Bootstrap resampling indicated variability in the data-derived optimal threshold, with a median selected threshold of 25 points and an approximate 95% range of 23–33 points. Therefore, the 25-point threshold should be regarded as exploratory and cohort-specific rather than as a validated clinical cut-off (Supplementary Table S1).

## 4. Discussion

The principal finding of this study is that higher FES-I scores were independently associated with the occurrence of at least one fall during two years of follow-up in patients with CKD, even after adjustment for selected clinical confounders. In the parsimonious multivariable model, each 1-point increase in FES-I was associated with approximately 15% higher odds of experiencing at least one fall. The findings suggest that FES-I may provide incremental prognostic information beyond a basic clinical model including age, sex, dialysis status, comorbidity, and functional status. Addition of FES-I increased the apparent AUC from 0.826 to 0.874 and significantly improved overall model fit in the likelihood-ratio test (χ^2^ = 15.45, df = 1, *p* = 0.000085). However, the difference in AUC did not reach the conventional threshold for statistical significance in the paired DeLong comparison (*p* = 0.0529). Following internal validation, the optimism-corrected AUCs were 0.798 and 0.850 for the basic and extended models, respectively. Therefore, the incremental predictive value of FES-I should be regarded as exploratory and requires confirmation in independent cohorts.

The present analysis does not establish a definitive clinical cut-off for FES-I. Although an analysis of the final outcome data identified 25 points as the threshold maximizing the Youden index. Moreover, bootstrap resampling demonstrated variability in the selected threshold. Consequently, any threshold derived from this relatively small single-center cohort should be considered exploratory and cohort-specific. Prospective external validation is required before a specific FES-I cut-off can be recommended for clinical decision-making.

Neurological and neuromuscular impairments also play a significant role. Progressive sensorimotor dysfunction in CKD patients contributes to impaired balance and coordination [9]. Reduced muscle strength and impaired physical function have been identified as key determinants of falls in hemodialysis populations [10]. These findings are consistent with the observed correlation between FES-I and objective measures of coordination in S-PPA tests. Although the present findings support an association between FES-I and objective measures of physical function, the FES-I also captures subjective perceptions of instability and confidence. Therefore, psychological, behavioral, and environmental factors may contribute to FES-I scores independently of physiological impairment.

Adverse outcomes, such as falls, hospitalization, and mortality, have been repeatedly linked to frailty, which is quite common in CKD patients [6,7]. Additionally, no disease-specific impairments such as reduced mobility, fatigue, and cognitive decline further contribute to increased vulnerability [8]. These factors may influence both objective fall risk and subjective perception of instability, which is captured by the FES-I. These findings suggest that higher FES-I scores may be associated with several dimensions of patient vulnerability, including functional, neurological, and potentially vascular factors. However, the present observational data do not establish the mechanisms underlying these associations.

In the present study, higher FES-I scores were associated with higher PWV values, and a moderate positive correlation between these variables was observed. However, this association was based on an unadjusted cross-sectional analysis and should therefore be interpreted with caution. Participants with higher FES-I scores were also older and had a greater burden of comorbidity, frailty, and cardiovascular risk factors (Table 1), all of which may have contributed to the observed relationship. Increased arterial stiffness, reflected by elevated PWV, is a well-established marker of cardiovascular risk, CKD progression, and mortality in patients with chronic kidney disease [13,15]. Moreover, vascular dysfunction has been associated with impaired cerebral perfusion, cognitive decline, and an increased risk of falls in older adults [17,18,19,20]. Nevertheless, our findings do not establish an independent association between FES-I and arterial stiffness, nor do they support a causal or mechanistic link between vascular dysfunction and fear of falling. Rather, they suggest that arterial stiffness may represent one of several factors associated with greater overall vulnerability in patients with CKD. Therefore, the observed relationship should be considered exploratory and hypothesis-generating. Future studies using multivariable analyses in larger cohorts are warranted to determine whether the association between FES-I and PWV remains independent after adjustment for age, comorbidity, frailty, CKD severity, and other relevant clinical confounders.

The risk of falls in CKD is significantly increased by visual impairment. Ocular conditions such as diabetic retinopathy and macular degeneration are more common in patients with chronic kidney disease (CKD) [21]. Impaired contrast sensitivity and visual function are associated with reduced mobility and postural stability [22]. Population-based studies have confirmed that visual impairment significantly increases fall risk [26]. Additionally, interactions between visual, cognitive, and motor systems may further exacerbate instability [27,28].

From a clinical perspective, FES-I may represent a simple and low-cost candidate screening instrument for identifying patients with CKD who may be at increased risk of falls. Its ease of administration may facilitate its use in routine nephrology practice; however, its incremental clinical utility and optimal threshold require prospective external validation before routine implementation can be recommended. If validated in future prospective studies, the FES-I may facilitate the identification of patients who could potentially benefit from individualized fall-prevention strategies, such as balance training, physical rehabilitation, medication review, management of visual impairment, or optimization of modifiable cardiovascular risk factors. However, the present study was not designed to evaluate the effectiveness of these interventions.

The present cohort included both non-dialysis CKD patients (stages G3–G5) and patients receiving maintenance hemodialysis. Owing to the limited sample size, stratified analyses according to dialysis status were not performed. Therefore, the present findings should be interpreted as applying to the overall CKD cohort rather than to individual CKD subgroups. Furthermore, because only independently ambulatory hospitalized patients were eligible for inclusion, the results should not be generalized to patients requiring walking aids or wheelchairs, who may represent a particularly high-risk subgroup for falls. Future research should assess the FES-I’s predictive performance independently in populations receiving dialysis and those not, as well as in patients with more severe mobility impairment.

This study’s prospective design, use of both subjective and objective fall risk assessments, and integration of vascular assessment with functional evaluation are among its strong points.

This study has a number of limitations that should be noted. The study population consisted exclusively of independently ambulatory patients with CKD. Individuals requiring walking aids or wheelchairs were excluded because several functional assessments used in this study required independent ambulation. Consequently, the findings cannot be generalized to patients with more advanced mobility impairment, who may represent an even higher-risk subgroup for falls. First, the study was conducted at a single center and included a relatively small sample, which may limit the generalizability of the findings. Second, falls were assessed retrospectively during the final follow-up visit rather than by prospective fall diaries or scheduled interim assessments. Consequently, recall bias cannot be excluded, particularly with respect to minor fall events that may not have been remembered by participants. Furthermore, outcome assessment was not performed under blinded conditions with respect to baseline FES-I results, which may have introduced observer bias. Residual confounding due to unmeasured factors, such as medication burden, cognitive impairment, or other clinical characteristics, also cannot be excluded. In addition, 14 of the 144 enrolled participants died before the 2-year follow-up assessment and could therefore not be included in the final analysis. Consequently, the primary analysis was restricted to the 130 participants for whom follow-up data were available. This may have introduced attrition bias, as participants who died during follow-up may have differed systematically from those who survived to the follow-up assessment. These limitations should be considered when interpreting the observed discriminatory performance of the FES-I and the proposed cut-off value. To increase the precision and thoroughness of fall ascertainment, future prospective studies should employ fall diaries or electronic reporting systems with planned intermediate follow-up.

## 5. Conclusions

Higher FES-I scores were independently associated with the occurrence of falls during two-year follow-up in this cohort of patients with CKD and may provide additional prognostic information beyond selected clinical risk factors. FES-I may therefore represent a simple candidate screening instrument for fall-risk assessment in CKD. However, the optimal threshold was not stable on internal validation, and the present findings, including any proposed cut-off, require prospective external validation before clinical adoption.

## Figures and Tables

**Table 1:** Exploratory comparison of demographic, clinical, functional, hemodynamic, and laboratory characteristics according to FES-I score (≤22 vs. >22 points).

	FES-I ≤ 22; N = 66	FES-I > 22; N = 64	*p*
**Demographic**			
Age (years)	57.16 ± 15.21	72.11 ± 9.22	<0.01
Female (%)	22 (32)	32 (50)	>0.05
Weight (kg)	77 ± 17.69	80.59 ± 19.41	>0.05
Height (m)	1.71 ± 0.09	1.66 ± 0.09	<0.01
BMI (m/kg^2^)	26.55 ± 5.03	29.1 ± 5.72	<0.01
**Cause of CKD**			
HTN and DM (%)	16 (25)	40 (63)	<0.05
GN (%)	26 (39)	11 (17)	<0.05
Others (%)	23 (35)	13 (20)	>0.05
Unknown cause, n (%)	1 (1)	0	
**Comorbidity**			
CCI (points)	4.23 ± 2.4	6.34 ± 1.98	<0.01
Number of HD patients (%)	19 (28)	21 (33)	>0.05
Smoking (pack-years)	6.71 ± 13.14	8.1 ± 15.51	>0.05
**Mobil-O-Graph^®^ blood pressure monitor parameters**			
pSBP (mmHg)	134.3 ± 21.11	144.91 ± 28.26	>0.05
pMBP (mmHg)	108.64 ± 16.46	110.98 ± 19.76	>0.05
pDBP (mmHg)	86.82 ± 16.28	82.25 ± 14.33	>0.05
pPP (mmHg)	46.43 ± 16.77	62.86 ± 20.74	<0.01
HR (beats/min)	76.29 ± 15.67	68.9 ± 12.39	<0.05
AIx@75 (%)	18.21 ± 13.47	20.88 ± 17.21	>0.05
Stroke Volume (mL)	70.31 ± 16.17	80.95 ± 17.69	<0.01
CO (L/min)	5.21 ± 0.99	5.46 ± 1.03	>0.05
Augmentation pressure (mmHg)	6.5 ± 6.18	13.02 ± 12.59	<0.01
PWV (m/s)	8.33 ± 2.12	10.74 ± 2.12	<0.01
**QRISK3—risk of having a heart attack or stroke within the next 10 years (%)**			
QRISK3 (%)	19.53 ± 13.43	34.43 ± 14.68	<0.01
Short Form Physiological Profile Assessment			
S-PPA (z score)	2.79 ± 2.09	4.51 ± 2.38	<0.01
Fall risk score (point)	3.52 ± 1.14	4.23 ± 0.76	<0.01
Melbourne Edge Test (dB)	16.47 ± 3.16	13.17 ± 3.98	<0.01
Proprioception (°)	0.92 ± 1.34	4.17 ± 6.84	<0.01
Isometric knee extension test right (kg)	23.78 ± 9.95	17.4 ± 8.39	<0.01
Isometric knee extension test left (kg)	23.16 ± 9.76	16.26 ± 8.96	<0.01
The hand reaction time test (ms)	285.46 ± 114.13	385.86 ± 160.3	<0.01
The postural sway test (cm^2^)	73.9 ± 148.2	166.96 ± 225.84	<0.01
30-Second Sit-to-Stand Test (No repetitions)	15.5 ± 6.53	8.12 ± 4.95	<0.01
**Laboratory**			
Hemoglobin (g/dL)	10.83 ± 1.91	10.62 ± 1.72	>0.05
Total Protein (g/dL)	6.19 ± 0.92	6.1 ± 0.73	>0.05
Serum Albumin (g/dL)	3.50 ± 0.58	3.33 ± 0.48	>0.05
Total Cholesterol (mg/dL)	208.14 ± 63.5	178.88 ± 66.02	<0.05
Triglycerides (mg/dL)	166.78 ± 82.18	153 ± 85.00	>0.05
CRP (mg/L)	12.78 ± 16.46	9.73 ± 14.65	>0.05
Ca++ (mg/dL)	8.86 ± 0.88	8.49 ± 0.9	>0.05
Pi (mg/dL)	5.79 ± 1.82	4.88 ± 1.02	<0.01
iPTH (pg/mL)	455 ± 492	339 ± 423	>0.05
AP (U/L)	145 ± 161	89 ± 55	<0.05
**Questionnaires**			
Barthel index (points)	99.54 ± 1.45	87.70 ± 15.63	<0.01
Number of patients with Bartel index < 80 points	1 (1)	14 (22)	<0.05
CFS Rockwood score	2.52 ± 0.9	3.79 ± 1.14	<0.01
SF-36, QoL index (points)	102.7 ± 15.63	80.42 ± 17.99	<0.01
**2-years follow-up**			
Total number of falls over a two-year period	11	73	<0.01
Patients with ≥1 fall during follow-up	8	41	<0.01

Abbreviation: FES-I, Falls Efficacy Scale-International, BMI, body mass index; TG, triglycerides; CRP, C-reactive protein; Hypertensive nephropathy; DM, diabetes mellitus; GN, glomerulonephritis; CCI, Charlson Comorbidity Index; SBP, systolic blood pressure; MBP, mean blood pressure; DBP, diastolic blood pressure; PP, pulse pressure; HR, heart rate; PWV, pulse wave velocity; AIx@75, augmentation index normalized to a heart rate of 75 beats/min; QRISK3, 10-year risk of cardiovascular accident; S-PPA, Short-Form Physiological Profile Assessment; CFS, Clinical Frailty Scale; SF-36, 36-Item Short Form Health Survey; QoL, Quality of Life; TC, total cholesterol; TG, triglycerides; CRP, C-reactive protein; Ca++, calcium; Pi, phosphate; PTH, parathormone; AP, alkaline phosphatase; sCr, serum creatinine; GFR, glomerular filtration rate. chi-square test.

**Table 2:** Multivariable logistic regression analysis of factors associated with at least one fall during the 2-year follow-up.

Variable	OR	95% CI	*p*
Age (years)	1.035	0.98–1.09	0.216
Sex (female)	2.437	0.88–6.78	0.088
Hemodialysis	2.177	0.77–6.15	0.142
Barthel scale (points)	1.050	0.976–1.130	0.190
CCI	1.149	0.898–1.472	0.269
FES-I (points)	1.145	1.06–1.24	< 0.001

Abbreviations: OR, odds ratio; CI, confidence interval; CCI, Charlson Comorbidity Index; FES-I, Falls Efficacy Scale-International. The complete-case model included 120 of 130 participants, including 44 of 49 participants who experienced at least one fall. Ten participants were excluded because of missing covariate data (current hemodialysis status, n = 9; Barthel scale, n = 1). All variables were entered simultaneously. No substantial multicollinearity was detected (all VIFs < 5; maximum VIF = 3.31).

**Table 3:** Classification of participants according to the exploratory FES-I threshold of ≥ 25 points.

	Fall During Follow-Up	No Fall During Follow-Up	Total
FES-I ≥ 25	40	21	61
FES-I < 25	9	60	69
Total	49	81	130

## Data Availability

The data presented in this study are available upon request from the corresponding author.

## Supplementary Materials

The following supporting information can be downloaded at: https://www.mdpi.com/article/10.3390/jcm15166455/s1, Table S1: Apparent and bootstrap-validated performance of the nested logistic regression models.

## References

[1] Lin P., Wan B., Zhong J., Wang M., Tang F., Wang L., Guo J., Ye Y., Liu X., Peng L. (2024). Risk of fall in patients with chronic kidney disease: Results from the China health and retirement longitudinal study (CHARLS). BMC Public Health.

[2] López-Soto P.J., De Giorgi A., Senno E., Tiseo R., Ferraresi A., Canella C., Rodríguez-Borrego M.A., Manfredini R., Fabbian F. (2015). Renal disease and accidental falls: A review of published evidence. BMC Nephrol..

[3] Bowling C.B., Booth J.N., Gutiérrez O.M., Tamura M.K., Huang L., Kilgore M., Judd S., Warnock D.G., McClellan W.M., Allman R.M. (2014). Nondisease-specific problems and all-cause mortality among older adults with CKD: The REGARDS Study. Clin. J. Am. Soc. Nephrol..

[4] Kistler B.M., Khubchandani J., Jakubowicz G., Wilund K., Sosnoff J. (2018). Falls and Fall-Related Injuries Among US Adults Aged 65 or Older with Chronic Kidney Disease. Prev. Chronic Dis..

[5] Li M., Tomlinson G., Naglie G., Cook W.L., Jassal S.V. (2008). Geriatric comorbidities, such as falls, confer an independent mortality risk to elderly dialysis patients. Nephrol. Dial. Transplant..

[6] Lord S.R., Menz H.B., Tiedemann A. (2003). A physiological profile approach to falls risk assessment and prevention. Phys. Ther..

[7] Zanotto T., Mercer T.H., van der Linden M.L., Rush R., Traynor J.P., Petrie C.J., Doyle A., Chalmers K., Allan N., Shilliday I. (2020). The relative importance of frailty, physical and cardiovascular function as exercise-modifiable predictors of falls in haemodialysis patients: A prospective cohort study. BMC Nephrol..

[8] Shirai N., Yamamoto S., Osawa Y., Tsubaki A., Morishita S., Sugahara T., Narita I. (2024). Low muscle strength and physical function contribute to falls in hemodialysis patients, but not muscle mass. Clin. Exp. Nephrol..

[9] Cook W.L., Tomlinson G., Donaldson M., Markowitz S.N., Naglie G., Sobolev B., Jassal S.V. (2006). Falls and Fall-Related Injuries in Older Dialysis Patients. Clin. J. Am. Soc. Nephrol..

[10] Bossola M., Marino C., Di Napoli A., Agabiti N., Tazza L., Davoli M. (2008). Functional impairment and risk of mortality in patients on chronic hemodialysis: Results of the Lazio Dialysis Registry. J. Nephrol..

[11] Doshi S., Moorthi R.N., Fried L.F., Sarnak M.J., Satterfield S., Shlipak M., Lange-Maia B.S., Newman A.B., Strotmeyer E.S. (2020). Chronic kidney disease as a risk factor for peripheral nerve impairment in older adults: A longitudinal analysis of Health, Aging and Body Composition (Health ABC) study. PLoS ONE.

[12] Papakonstantinopoulou K., Sofianos I. (2017). Risk of falls in chronic kidney disease. J. Frailty Sarcopenia Falls.

[13] Zhang F., Wang H., Bai Y., Zhang Y., Huang L., Zhang H. (2023). Prevalence of physical frailty and impact on survival in patients with chronic kidney disease: A systematic review and meta-analysis. BMC Nephrol..

[14] Mei M.F., Gao M.Q., Chen M.F., Zhao M.L., Shang B.Y., Hu M.K., Zhang M.W., Zhao M.B. (2021). Frailty as a Predictor of Negative Health Outcomes in Chronic Kidney Disease: A Systematic Review and Meta-Analysis. J. Am. Med. Dir. Assoc..

[15] Zanoli L., Lentini P., Briet M., Castellino P., House A.A., London G.M., Malatino L., McCullough P.A., Mikhailidis D.P., Boutouyrie P. (2019). Arterial Stiffness in the Heart Disease of CKD. J. Am. Soc. Nephrol. JASN.

[16] Zuo J., Hu Y., Chang G., Chu S.L., Tan I., Butlin M., Avolio A. (2020). Relationship between Arterial Stiffness and Chronic Kidney Disease in Patients with Primary Hypertension. J. Hum. Hypertens..

[17] Chandra P., Sands R.L., Gillespie B.W., Levin N.W., Kotanko P., Kiser M., Finkelstein F., Hinderliter A., Rajagopalan S., Sengstock D. (2014). Relationship between heart rate variability and pulse wave velocity and their association with patient outcomes in chronic kidney disease. Clin. Nephrol..

[18] Townsend R.R., Anderson A.H., Chirinos J.A., Feldman H.I., Grunwald J.E., Nessel L., Roy J., Weir M.R., Wright J.T., Bansal N. (2018). Association of Pulse Wave Velocity with Chronic Kidney Disease Progression and Mortality. Hypertension.

[19] Zanotto T., Mercer T.H., van der Linden M.L., Traynor J.P., Doyle A., Chalmers K., Allan N., Shilliday I., Koufaki P. (2020). Association of postural balance and falls in adult patients receiving haemodialysis: A prospective cohort study. Gait Posture.

[20] Karasavvidou D., Boutouyrie P., Kalaitzidis R., Kettab H., Pappas K., Stagikas D., Antonakis N., Tsalikakis D., Elisaf M., Laurent S. (2018). Arterial Damage and Cognitive Decline in Chronic Kidney Disease Patients. J. Clin. Hypertens..

[21] Aimagambetova B., Ariko T., Merritt S., Rundek T. (2024). Arterial stiffness measured by pulse wave velocity correlated with cognitive decline in hypertensive individuals: A systematic review. BMC Neurol..

[22] Wong A.K., Lord S.R., Trollor J.N., Sturnieks D.L., Delbaere K., Menant J., Brodaty H., Sachdev P.S., Close J.C. (2014). High Arterial Pulse Wave Velocity Is a Risk Factor for Falls in Community-Dwelling Older People. J. Am. Geriatr. Soc..

[23] Shenkin S.D., Rivers C.S., Deary I.J., Starr J.M., Wardlaw J.M. (2009). Maximum (prior) brain size, not atrophy, correlates with cognition in community-dwelling older people: A cross-sectional neuroimaging study. BMC Geriatr..

[24] Delbaere K., Close J.C., Mikolaizak A.S., Sachdev P.S., Brodaty H., Lord S.R. (2010). The Falls Efficacy Scale-International: A comprehensive validation study. Age Ageing.

[25] Zak M., Makara-Studzińska M., Mesterhazy A., Mesterhazy J., Jagielski P., Januszko-Szakiel A., Sikorski T., Jaworski P., Miszczuk R., Brola W. (2022). Validation of FES-I and Short FES-I Scales in the Polish Setting as the Research Tools of Choice to Identify the Fear of Falling in Older Adults. Int. J. Environ. Res. Public Health.

[26] Thompson A.C., Chen H., Miller M.E., Webb C.C., Williamson J.D., Marsh A.P., Hugenschmidt C.E., Baker L.D., Laurienti P.J., Kritchevsky S.B. (2023). Association Between Contrast Sensitivity and Physical Function in Cognitively Healthy Older Adults: The Brain Networks and Mobility Function Study. J. Gerontol. Ser. A.

[27] Allali G., Launay C.P., Blumen H.M., Callisaya M.L., De Cock A.M., Kressig R.W., Srikanth V., Steinmetz J.P., Verghese J., Beauchet O. (2017). Falls, Cognitive Impairment, and Gait Performance: Results from the GOOD Initiative. J. Am. Med. Dir. Assoc..

[28] Wood J.M., Lacherez P.F., Black A.A., Cole M.H., Boon M.Y., Kerr G.K. (2009). Postural Stability and Gait among Older Adults with Age-Related Maculopathy. Investig. Ophthalmol. Vis. Sci..

